# Intermittent theta-burst stimulation promotes neurovascular unit remodeling after ischemic stroke in a mouse model

**DOI:** 10.4103/NRR.NRR-D-24-01189

**Published:** 2025-03-25

**Authors:** Jingjun Zhang, Ming Ding, Lu Luo, Dan Huang, Siyue Li, Shuying Chen, Yunhui Fan, Li Liu, Hongyu Xie, Gang Liu, Kewei Yu, Junfa Wu, Xiao Xiao, Yi Wu

**Affiliations:** 1 Department of Rehabilitation Medicine, Huashan Hospital, Fudan University, Shanghai, China; 2 Department of Rehabilitation Medicine, The Sixth People’s Hospital Affiliated to Shanghai Jiao Tong University School of Medicine, Shanghai, China; 3 Behavioral and Cognitive Neuroscience Center, Institute of Science and Technology for Brain-Inspired Intelligence, Fudan University, Shanghai, China; 4 Department of Laboratory Medicine, Huashan Hospital, Fudan University, Shanghai, China

**Keywords:** astrocyte, blood–brain barrier, glycogen synthase kinase 3 beta, intermittent theta-burst stimulation, ischemic stroke, microglia, neural protection, neuroinflammation, neurovascular unit, vascular protection

## Abstract

The neurovascular unit plays a critical role in maintaining brain structure, function, and homeostasis. Following ischemic stroke, dysfunction and dysregulation of this unit contribute to nerve–blood vessel uncoupling. Intermittent theta-burst stimulation is a repetitive transcranial magnetic stimulation that operates within the theta wave range and can either promote or inhibit cortical excitability. Previous studies have shown that intermittent theta wave stimulation has neuroprotective effects, but the underlying mechanisms remain unclear. In this study, mice subjected to middle cerebral artery occlusion/reperfusion were treated with intermittent theta-burst stimulation. The results showed that intermittent theta-burst stimulation significantly improved neurological function and motor recovery, reduced apoptosis in the peri-infarct region, and activated the PI3K/AKT/GSK3β/β-catenin signaling pathway. Additionally, intermittent theta-burst stimulation suppressed inflammation through the PI3K/AKT/GSK3β and NF-κB pathways. Notably, intermittent theta-burst stimulation strengthened A2 astrocyte–blood vessel coupling, and the effects of intermittent theta-burst stimulation were reversed by the PI3K inhibitor LY294002. These findings demonstrate that intermittent theta-burst stimulation promotes neurovascular unit remodeling and improves neurological outcomes by modulating microglia and astrocytes via the PI3K/AKT/GSK3β and NF-κB signaling pathways.

## Graphical abstract

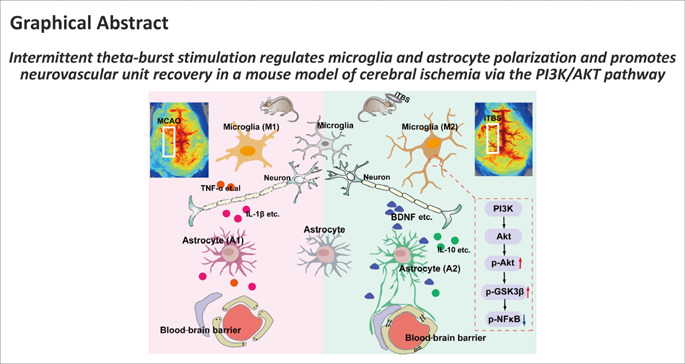

## Introduction

Stroke is the second most common cause of death worldwide, and is linked to high rates of morbidity, mortality, and disability (Krishnamurthi et al., 2020). Notably, ischemic stroke (IS) accounts for about 80% of stroke cases, and is associated with more deaths and disability-adjusted life-years lost than other forms of stroke (Donkor, 2018; Katan and Luft, 2018). Despite the availability of numerous clinical interventions (e.g., thrombolytic and endovascular thrombectomy) for treatment and prevention, many patients either die or face enduring severe disabilities after experiencing IS (Campbell et al., 2019; Mosconi and Paciaroni, 2022). Moreover, the efficacy of secondary prevention is impeded by limited medication choices and low patient adherence to prescribed treatments (Mosconi and Paciaroni, 2022). Neuroprotective agents, including stem cells, are being developed in animal models of IS, but have not shown satisfactory clinical outcomes (Mosconi and Paciaroni, 2022). Therefore, identifying effective rehabilitation strategies for brain injury caused by IS is crucial, particularly in patients with chronic symptoms (Schmidt and Minnerup, 2016; Herpich and Rincon, 2020).

A clinical study showed that, among patients who undergo endovascular treatment and achieve complete reperfusion, more than one-third experience poor outcomes (Mujanovic et al., 2024) that are closely associated with the severity of cerebral ischemia, collateral circulation, neuroinflammation, and the status of microvascular perfusion (Jurcau and Simion, 2021). This underscores that, once cerebral ischemia occurs, it indiscriminately damages all cells and tissues, and solely relying on neuroprotective treatments is unlikely to alter stroke outcomes (Palomares and Cipolla, 2011). Therefore, comprehensive brain protection is crucial for improving the outcomes of reperfusion injury after stroke. Ischemic reperfusion disrupts autoregulation of blood flow in the cerebral arteries (Euser and Cipolla, 2007), leading to intracranial hypertension in approximately 40%–50% of patients within 3 days of stroke (Olsen et al., 1981).

While neuroprotective drugs have shown promise in animal models of stroke, many have failed in clinical trials for IS. One key limitation is disruption of the blood–brain barrier during the acute phase, caused by free radicals and matrix metalloproteinase 9, leading to impaired neuronal function. In this context, the neurovascular unit (NVU), which comprises a network of neurons, astrocytes, and vascular endothelial cells, has been suggested as a novel treatment target for stroke (Iadecola, 2017). Studies indicate that changes in the concentrations of different molecular components (e.g., glial proteins, amino acid neurotransmitters, inflammatory substances, and neurotrophic factors) within the NVU are closely linked to IS stages, the extent of brain injury, and patients’ neurological status (Steliga et al., 2020). NVU impairment and disruption are crucial to neurovascular decoupling, which affects both neuronal elements and the vascular system (Yu et al., 2020; Shang et al., 2021). The NVU initiates a coordinated response to sustain and restore blood flow, aiming to mitigate neuronal damage (Forró et al., 2021). Hence, promoting NVU functional recovery is essential for the effective management of IS.

Post-stroke rehabilitation is integral to recovery, emphasizing relearning lost skills and achieving maximum independence. Innovative approaches, such as repetitive transcranial magnetic stimulation (rTMS), a noninvasive method of brain stimulation, have recently been developed for addressing neurological and psychiatric disorders (Starosta et al., 2022). Increasing evidence suggests two models of neuroplastic reorganization in stroke recovery: one highlights an imbalance that leads to heightened excitability in the unaffected hemisphere, while the other proposes that activity in this hemisphere is centered on compensatory mechanisms (Dionísio et al., 2018). rTMS therapy is painless and can influence cortical excitability not only in the targeted area but also in distant regions (Fisicaro et al., 2019). Low frequency rTMS (less than 1 Hz) decreases cortical excitability, while high frequency rTMS (greater than 5 Hz) increases it (Fisicaro et al., 2019; Lefaucheur et al., 2020; Turi et al., 2021). Intermittent theta-burst stimulation (iTBS) is a type of rTMS that operates within the theta wave range and can be used to either promote or inhibit cortical excitability (Dionísio et al., 2018).

iTBS is a patterned form of rTMS that employs clustered bursts of stimulation (Oberman et al., 2011), with each cluster equivalent to a single pulse of conventional rTMS. When multiple clusters are combined, they mimic a continuous sequence of stimulation typical of traditional rTMS. Compared with conventional rTMS, iTBS offers advantages such as reduced stimulation duration, lower intensity, prolonged effects, and greater alignment with the physiological state of neural activity (Lehmann et al., 2002; Cole et al., 2024). Clinical and animal studies have demonstrated that iTBS is generally safe and well tolerated (Yegiyants et al., 2010; Somaa et al., 2022), with adverse effects predominantly consisting of temporary dizziness or scalp discomfort, while severe side effects, such as seizures, are extremely rare. In animal models, the incidence of adverse reactions is closely related to stimulation parameters, including intensity, frequency, and duration (Uzair et al., 2022). Long-term interventions using iTBS have not shown significant neurological damage or serious adverse effects. Multiple studies have shown that iTBS has neuroprotective effects (Ba et al., 2019; Chen et al., 2019), enhances neurological function (Zong et al., 2020), inhibits inflammation (Luo et al., 2022a), improves cerebral blood flow (CBF) (Park et al., 2017; Iyer and Madhavan, 2018), and promotes angiogenesis (Luo et al., 2017; Lee et al., 2022) in animal models of brain injury. However, the exact cellular targets and the molecular mechanisms underlying the effects of iTBS remain unclear (Uzair et al., 2022).

Following IS, NVU regeneration and repair are crucial for functional remodeling (Hatakeyama et al., 2020). The phosphoinositide 3-kinase/protein kinase B (PI3K/AKT) and Wnt/β-catenin pathways are essential in neurovascular remodeling (Samakova et al., 2019; Song et al., 2021). These pathways facilitate neuronal and blood vessel regeneration by modulating glycogen synthase kinase 3 beta (GSK3β) phosphorylation and inactivation and by facilitating nuclear expression of β-catenin. GSK3β, a significant IS treatment target, is expressed at high levels throughout the nervous system (Wang et al., 2017). Moreover, brain-derived neurotrophic factor (BDNF) regulates GSK3β expression through the PI3K/AKT signaling pathway (Hetman et al., 1999; Gupta et al., 2014). Remarkably, rTMS has been shown to significantly enhance BDNF expression in both the cortex and hippocampus, as well as activate the PI3K/AKT signaling pathway in neural cells (Liu et al., 2020b). Moreover, extensive evidence shows that microglia and astrocytes are crucial targets for facilitating NVU remodeling in IS (Liu et al., 2020a; Williamson et al., 2021), iTBS enhances this process by modulating microglial and astrocyte polarization (Ferreira et al., 2024), suggesting possible connections among rTMS, the PI3K/AKT pathway, astrocytes, and neurovascular remodeling after IS.

In this study we used proteomics analysis, behavioral tests, molecular biology, and immunohistochemistry to determine the effects of iTBS on neurovascular remodeling after IS and to investigate the underlying mechanism.

## Methods

### Animals

Male, specific pathogen–free C57BL/6 mice (aged 7–8 weeks, weighing 20–25 g) were purchased from Shanghai Jihui Laboratory Animal Care Co., Ltd. (Shanghai, China; license No. SCXK (Hu) 2017-0012). Only male mice were used to ensure consistent experimental conditions and procedural feasibility, while eliminating the potential impact of cyclic estrogen fluctuations in female mice on the stroke model and overall experimental outcomes (Koellhoffer and McCullough, 2013). All the animals were housed in an environment with a temperature of 22 ± 1°C, a relative humidity of 50% ± 1%, and a 12/12-hour light/dark cycle. All procedures involving animals (including euthanasia) were conducted in compliance with the regulations and guidelines of Fudan University Institutional Animal Care, the Association for Assessment and Accreditation of Laboratory Animal Care, the Institutional Animal Care and Use Committee, and the National Institutes of Health Guide for the Care and Use of Laboratory Animals. All efforts were made to provide the animals with good care and alleviate their pain. All experiments described in this study were approved by the Animal Care and Use Committee of Huashan Hospital Affiliated with Fudan University (approval No. 2021JS Huashan Hospital-128; February 27, 2021).

### Focal cerebral ischemia–reperfusion

Focal cerebral ischemia–reperfusion was induced by middle cerebral artery occlusion (MCAO), as described by Luo et al. (2022b). Mice were anesthetized with 1% pentobarbital sodium (100 mg/kg; Sigma-Aldrich, St. Louis, MO, USA) by intraperitoneal injection and maintained at 37.0 ± 0.3°C using a heating pad. A midline incision was made to expose the left common carotid artery, external carotid artery, and internal carotid artery. The common carotid artery and internal carotid artery were ligated, and a 6-0 coated filament (602156PK; Doccol Corp., Cambridge, MA, USA) was introduced through the external carotid artery. The filament was advanced into the internal carotid artery to occlude the middle cerebral artery and left in place for 60 minutes. After occlusion, the filament and ligature were removed to allow reperfusion. Then, the incision was sutured, bupivacaine was applied for pain relief, and the mouse was placed in a preheated cage to recover.

### Cerebral blood flow detection

Regional CBF was monitored using laser speckle flowmetry (RWD Life Science, Shenzhen, China). Mice were anesthetized with 1% pentobarbital sodium by intraperitoneal injection and secured on a stereotaxic frame (RWD Life Science). A midline scalp incision was made to expose the skull, which was kept moist with saline. Laser speckle imaging parameters, including image processing mode, spatial filtering constant, frame rate, and recording mode, were set. CBF measurements were recorded on the left hemisphere before ischemia, 10 minutes after ischemia, and 10 minutes after reperfusion.

### Modified neurological severity score

Twenty-four hours post-MCAO/reperfusion (MCAO/r) surgery, neurological deficits were assessed using the modified neurological severity score (mNSS), which evaluates motor function, reflexes, and balance, with a score range of 0 to 14 (Luo et al., 2022b). Mice were given one point for each test that they failed to complete. Mice with an mNSS score ≥ 6 were included in the experiment. Sham-operated animals underwent the same anesthetic and surgical procedures, except that the middle cerebral artery was not occluded.

### Repetitive transcranial magnetic stimulation

A customized magnetic stimulator (CCY-II, Wuhan Yiruide Medical Equipment, Wuhan, China) connected to a round coil (64 mm diameter, 3.46 T peak magnetic output) was used to stimulate MCAO/r mice. Motor-evoked potentials in the contralateral gastrocnemius muscle were recorded with needle electromyography electrodes. The stimulation intensity was increased by 5% until the resting motor threshold was reached, defined as the minimum output that elicited a muscle twitch or motor-evoked potential with a peak-to-peak amplitude > 50 μV in at least 5 out of 10 trials. The average resting motor threshold was 25% of the maximum output (ranging from 20% to 30%). The stimulation intensity for iTBS was set at 100% of the average resting motor threshold. Motor thresholds were checked randomly at the start of each stimulation session. In the formal experiment, iTBS (10 bursts of 50 Hz with three pulses each, repeated 20 times at 5-Hz intervals) was applied twice daily (9:00 a.m. to 11:00 a.m. and 4:00 p.m. to 6:00 p.m.), starting 48 hours post-MCAO/r injury for 7 consecutive days. The Sham and MCAO/r groups underwent the same protocol, with the coil positioned 15 cm above the head. Prior to the experiment, each mouse underwent grip training. iTBS was delivered while the mice were awake.

### Animal grouping and drug administration

We conducted proteomics analysis of the following groups: the (1) Sham group (*n* = 8); (2) MCAO/r group (*n* = 8); and (3) MCAO/r + iTBS group (*n* = 8). We conducted mechanistic studies using the following groups: the (1) Sham group (*n* = 15); (2) MCAO/r group (*n* = 25); (3) MCAO/r + iTBS group (*n* = 25); and (4) MCAO/r + iTBS + LY294002 (40 mg/kg, intraperitoneal, once a day; MCE, Shanghai, China, Cat# HY-10108) group (*n* = 25). LY294002 is a classic PI3K inhibitor. The optimal dose of LY294002 was determined according to the instruction manual and previously published articles (Lu et al., 2014; Fei et al., 2021).

### Behavioral tests

Behavioral tests were performed 1 day before and 9 days after MCAO/r surgery. The interval between each behavioral test was at least 2 hours, and the behavioral tests were carried out in the following order: (1) open field test, (2) cylinder test, and (3) Catwalk XT gait test.

#### Open field test

The open field test was used to assess exploration behavior in MCAO/r mice. The apparatus consisted of a white plastic box (40 cm × 40 cm × 50 cm) and a tracking system (EthovisionXT15, Nuldus, Wageningen, the Netherlands). After adjusting the system settings, the mouse was placed at the center of the box, and its movement trajectory within the 5-minute test period was recorded. The total distance traveled and average speed were analyzed using the Ethovision system (Luo et al., 2021).

#### Cylinder test

The cylinder test was used to assess forelimb asymmetry in MCAO/r mice. The apparatus consisted of a transparent cylinder (8 cm in diameter, 15 cm heigh), two mirrors, and a camera with slow-motion, high-definition recording. The mouse was placed inside, and the number of times each forelimb (left, right, or both) touched the cylinder wall within 5 minutes was recorded. The laterality index was calculated using the formula: laterality index (%) = (left + 0.5 × both)/(right + left + both) × 100 (Zhang et al., 2019).

#### Catwalk gait test

Gait was assessed using the Catwalk XT animal gait analysis system (Nuldus), which includes a high-speed camera beneath a glass runway that records various gait parameters as the mice pass through. Mice underwent 1 week of adaptive training prior to the experiment. Testing was conducted in a quiet, dark room, with each mouse completing at least three runs. The following parameters were analyzed: gait duration, time spent standing still, limb swing time and speed, gait cycle period, average running speed, stride length, and step frequency (Caballero-Garrido et al., 2017).

### Tissue processing

Animal specimens were collected for western blotting, 2,3,5-triphenyltetrazolium chloride (TTC) staining, Nissl staining, TdT-mediated dUTP nick-end labeling (TUNEL), and immunofluorescence. The mice were euthanized through intraperitoneal injection of pentobarbital sodium at a dosage of 100–150 mg/kg. For western blotting, phosphate-buffered saline (PBS) was perfused through the heart, and brain tissue was extracted, sectioned coronally to target the infarction area, and then quickly frozen in liquid nitrogen and stored at –80°C. For TTC staining, PBS was perfused through the heart, and brain tissue was extracted. For Nissl, TUNEL, and immunofluorescence staining, PBS was used for cardiac perfusion, followed by fixation with 4% paraformaldehyde. Brain tissues were fixed at 4°C for 24 hours, then dehydrated with 10%, 20%, and 30% sucrose, embedded in optimal cutting temperature (OCT) compound, and stored at –80°C.

### Western blot assay

Total protein was extracted from mouse brain tissue by adding 150 µL of RIPA lysis buffer for every 20 mg of tissue. After homogenizing the mixture, it was incubated on ice for 30 minutes to ensure complete lysis. The sample was then centrifuged at 13,523 × *g* for 10 minutes at 4°C, and the supernatant was collected. Finally, the protein concentration in the supernatant was determined using a BCA Protein Assay Kit (Beyotime, Shanghai, China). Western blotting for brain tissue proteins was performed as previously described (Luo et al., 2022b). Briefly, 30 μg of denatured protein was separated by constant voltage (150 V) electrophoresis on a sodium dodecyl sulfate polyacrylamide gel electrophoresis gel (10%, Epizyme Biotech, Shanghai, China) and then transferred to a polyvinylidene fluoride membrane (Millipore, Billerica, MA, USA) by constant current (280 MA). After blocking with 5% bovine serum albumin (Epizyme Biotech) for 1 hour at room temperature, the membrane was incubated overnight at 4°C with the following primary antibodies: anti-PI3K (rabbit, 1:1000, Affinity, Shanghai, China, Cat# AF6241, RRID: AB_2835340), anti-AKT (rabbit, 1:1000, Affinity, Cat# AF6261, RRID: AB_2835121), anti-GSK3β (rabbit, 1:1000, Affinity, Cat# AF5016, RRID: AB_2834935), anti-phospho-AKT[Ser473] (rabbit, 1:1000, Affinity, Cat# AF0016, RRID: AB_2810275), anti-phospho-GSK3β[Ser9] (rabbit, 1:1000, Affinity, Cat# AF2016, RRID: AB_2834439), anti-β-catenin (rabbit, 1:5000, Proteintech, Wuhan, China, Cat# 51067-2-AP, RRID: AB_2086128), anti-BDNF (rabbit, 1:1000, Affinity, Cat# DF6387, RRID: AB_2838350), anti-zonula occludens protein 1 (ZO-1; rabbit, 1:1000, Affinity, Cat# AF5145, RRID: AB_2837631), anti-occludin (rabbit, 1:1000, Affinity, Cat# DF7504, RRID: AB_2841004), anti-claudin-5 (rabbit, 1:1000, Affinity, Cat# AF5216, RRID: AB_2837702), anti-β-actin (mouse, 1:5000, Affinity, Cat# T0022, RRID: AB_2839417), anti-β-actin (rabbit, 1:5000, Affinity, Cat# AF7018, RRID: AB_2839420), anti-Bcl-2 (rabbit, 1:2000, Proteintech, Cat# 12789-1-AP, RRID: AB_2227948), anti-Bax (mouse, 1:5000, Proteintech, Cat# 60267-1-Ig, RRID: AB_2848213), anti-caspase 3 (rabbit, 1:1000, Proteintech, Cat# 19677-1-AP, RRID: AB_10733244), anti-vascular endothelial growth factor (VEGF; rabbit, 1:1000, Proteintech, Cat# 19003-1-AP, RRID: AB_2212657), anti-glial fibrillary acidic protein (GFAP; mouse, 1:2000, Proteintech, Cat# 60190-1-Ig, RRID: AB_10838694), anti-interleukin (IL)-1β (mouse, 1:1000, Proteintech, Cat# 66737-1-Ig, RRID: AB_2882087), anti-tumor necrosis factor alpha (TNF-α; mouse, 1:1000, Proteintech, Cat# 60291-1-Ig, RRID: AB_2833255), anti-IL-10 (mouse, 1:2000, Proteintech, Cat# 60269-1-Ig, RRID: AB_2881389), anti-nuclear factor kappa B (NF-κB; rabbit, 1:1000, Cell Signaling Technology, Danvers, MA, USA, Cat# 8242, RRID: AB_10859369), anti-phosphorylated NF-κB (p-NF-κB; rabbit, 1:1000, Cell Signaling Technology, Cat# 3033, RRID: AB_331284), anti-hypoxia-inducible factor 1α (HIF-1α; rabbit, 1:1000, Cell Signaling Technology, Cat# 36169, RRID: AB_2799095), anti-C3d (goat, 1:1000, R&D Systems, Minneapolis, MN, USA, Cat# AF2655, RRID: AB_2066622), and anti-S100A10 (rabbit, 1:1000, Proteintech, Cat# 11250-1-AP, RRID: AB_2269906). After the primary antibody incubation was completed, the polyvinylidene fluoride membrane was washed with Tris-buffered saline with Tween 20 (10 mM Tris, 150 mM NaCl, 0.05% Tween-20, pH 7.5) four times for 5 minutes each, and then incubated with horseradish peroxidase (HRP)-conjugated goat anti-mouse IgG (1:5000, Affinity, Cat# S0002, RRID: AB_2839430), goat anti-rabbit IgG (1:5000, Affinity, Cat# S0001, RRID: AB_2839429), or rabbit anti-goat IgG (1:5000, Affinity, Cat# S0010, RRID: AB_2844795) secondary antibody at room temperature (20–25°C) for 1.5 hours. The signals were imaged using an enhanced chemiluminescence developing solution (4a Biotech, Suzhou, China) and an ultraviolet gel imaging system (Tanon, Shanghai, China). The optical intensity was quantified using ImageJ software (version 1.50i, National Institutes of Health, Bethesda, MA, USA) (Schneider et al., 2012) and normalized to the loading control (β-actin).

### Nissl staining

Nissl staining was performed to assess neuronal damage in the peri-infarct region of MCAO/r mice. Frozen brain sections were sequentially dehydrated with 70%, 95%, and 100% alcohol solutions for 5 minutes each, followed by washing with 1× PBS three times for 5 minutes. After removing excess moisture, the sections were incubated in tar violet staining solution (Solarbio, Beijing, China) at 37°C for 5 minutes in the dark. The sections were then washed with double-distilled water, placed in Nissl’s differentiation solution for 3 minutes, and fixed in absolute ethanol for 5 minutes. After drying at room temperature, the sections were sealed with neutral resin and observed using the Olympus VS200 microscope (Olympus Optical, Ltd., Tokyo, Japan).

### Immunofluorescence staining and TdT-mediated dUTP nick end-labeling staining

Coronal sections (30-µm-thick) were taken from frozen brain tissue, and the sections surrounding the peri-infarct area were subjected to immunofluorescence staining and imaging analysis. First, the sections were washed three times for 5 minutes each with 1× PBS. Next, the 2- to 3-mm outer contours of the brain sections were incubated with a general-purpose antibody diluent containing 0.3% TritonX-100 (Sigma-Aldrich) for 20 minutes at room temperature followed by 1 hour of incubation at room temperature with bovine serum albumin. Next, the sections were incubated overnight at 4°C with the following primary antibodies: anti-phospho-AKT [Ser473] (rabbit, 1:300, Affinity, Cat# AF0016, RRID: AB_2810275), anti-phospho-GSK3β[Ser9] (rabbit, 1:300, Affinity, Cat# AF2016, RRID: AB_2834439), anti-NeuN (mouse, 1:300, Abcam, Cambridge, UK, Cat# ab104224, RRID: AB_10711040), anti-NeuN (rabbit, 1:300, Proteintech, Cat# 26975-1-AP, RRID: AB_2880708), anti-BDNF (rabbit, 1:300, Affinity, Cat# DF6387, RRID: AB_2838350), anti-BCL-2 (rabbit, 1:200, Proteintech, Cat# 12789-1-AP, RRID: AB_2227948), anti-C3d (goat, 1:200, R&D Systems, Cat# AF2655, RRID: AB_2066622), anti-S100A10 (rabbit, 1:200, Proteintech, Cat# 11250-1-AP, RRID: AB_2269906), anti-GFAP (rabbit, 1:300, Proteintech, Cat# 16825-1-AP, RRID: AB_2109646), anti-GFAP (mouse,1:300, Cell Signaling Technology, Cat# 3670, RRID:AB_561049), anti-Iba1 (rabbit, 1:300, FUJIFILM, Wako, Japan, Cat# 019-19741, RRID: AB_839504), anti-Iba1 (mouse,1:300, FUJIFILM, Cat# 011-27991, RRID: AB_2935833), and anti-CD31 (goat, 1:200, R&D Systems, Cat# AF3628, RRID: AB_2161028). After primary antibody incubation, the sections were washed with 1× PBS three times for 5 minutes each and incubated with Alexa Fluor 488 (goat, 1:1000, Abcam, Cat# ab150077, RRID: AB_2630356), Alexa Fluor 594 (goat, 1:1000, Abcam, Cat# ab150116, RRID: AB_2650601), and Alexa Fluor 647 (donkey, 1:1000, Abcam, Cat# ab150131, RRID: AB_2732857) secondary antibody at room temperature for 1.5 hours. Neuronal apoptosis in the peri-infarct area was assessed by TdT-mediated dUTP terminal labeling (TUNEL) assay (Solarbio) using a procedure adapted from previous studies (Fan et al., 2018; Luo et al., 2022b). After washing with PBS, the sections were mounted on slides using anti-fluorescence attenuation mounting medium containing 4′,6-diamidino-2-phenylindole (DAPI) (Solarbio). The brain sections were observed using an Olympus Fluorview-3000 confocal microscope (Olympus Optical, Ltd., Tokyo, Japan). Three distinct fields of view from the peri-infarct region of each brain section were selected for each mouse, TUNEL-positive cells were counted in each field of view, and the average value from all three images was calculated. Quantitative analysis of the immunofluorescence intensity in each experimental group was performed using ImageJ software.

### Proteomics analysis

Data preprocessing and postprocessing were performed using the Firmiana platform and R version 4.3 (Firmiana, Shanghai, China), respectively. The fraction of each protein out of the total protein was calculated, and differentially expressed proteins were identified on the basis of |fold change| > 2 and statistical significance (*P* < 0.05) using R. Functional enrichment analysis, including Gene Ontology and Kyoto Encyclopedia of Genes and Genomes analyses, was conducted using the R package Cluster Profiler to identify significant terms and pathways (adjusted *P* < 0.05).

### 2,3,5-Triphenyltetrazolium chloride staining

For infarct size detection, MCAO/r mice were anesthetized with 1% pentobarbital sodium, cardiac perfusion with pre-cooled 1× PBS was carried out, and the brains were removed. The brains were sliced coronally into 2-mm-thick sections and stained with 2% TTC solution (Solarbio) at 37°C for 20 minutes in the dark, then fixed in 4% paraformaldehyde. Unstained areas (white) were considered ischemic lesions. The infarct and hemisphere areas were quantified using ImageJ software. The edema index was calculated by dividing the left hemisphere area by the right hemisphere area, and the edema-adjusted infarct size was determined by dividing the infarct size by the edema index.

### Statistical analysis

Statistical analysis and data graphing were performed using GraphPad Prism 9.5.0 software (GraphPad Software, Boston, M, USA, www.graphpad.com). The data are presented as mean ± standard error of the mean (SEM). Differences between groups were analyzed using one-way or two-way analysis of variance for multiple comparisons followed by Tukey’s or Bonferroni’s *post hoc* test, and unpaired *t*-test was used for statistical analysis between two groups. *P* < 0.05 indicated a statistically significant difference.

## Results

### Intermittent theta-burst stimulation enhances peri-infarct cerebral perfusion and reduces infarct size in ischemic mice

**Figure 1:**
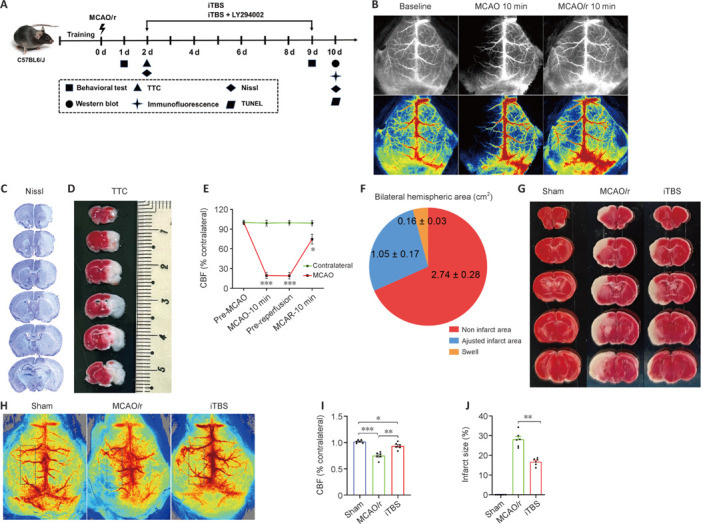
Study design and construction of a mouse model of cerebral ischemia. (A) Study design. (B) Laser speckle imaging of cerebral blood flow in mice: images of changes in cortical blood flow in mice before surgery (baseline), during middle cerebral artery occlusion (MCAO 10 minutes), and after removal of the occlusion and reperfusion (MCAO/r 10 minutes). Compared with the contralateral hemisphere, CBF in the infarcted side significantly decreased 10 minutes after thrombus insertion. (C) Nissl staining of mouse brain tissue 48 hours after MCAO/r. (D) TTC staining of mouse brain tissue 48 hours after MCAO/r; the unstained areas (white) were considered ischemic lesions. (E) Bilateral cortical blood flow before occlusion, 10 minutes after occlusion, and 10 minutes after reperfusion in the MCAO/r model. Compared with the contralateral side, cerebral blood flow decreased by more than 70% during infarction (*n* = 6). (F) Statistical analysis of the size of the infarct area in this cerebral ischemia model (*n* = 5). (G) TTC staining was used to assess the brain infarct area in the Sham, MCAO/r, and iTBS groups; unstained areas (white) were considered ischemic lesions. Compared with the MCAO/r group, iTBS significantly reduced the infarct size in the mouse model of cerebral ischemia. (H) Cerebral blood flow in the peri-infarct area was measured in the Sham, MCAO/r, and iTBS groups using laser speckle flowmetry. Compared with the MCAO/r group, iTBS significantly enhanced cerebral blood flow on the infarcted side. (I, J) Statistical analysis of cerebral blood flow and brain infarct area in each experimental group. Data are presented as mean ± SEM. *P < 0.05, **P < 0.01, ***P < 0.001 (one-way analysis of variance followed by Tukey’s multiple comparison test [I, J] or two-way analysis of variance followed by Bonferroni’s multiple comparison test [E]). CBF: Cerebral blood flow; iTBS: intermittent theta burst stimulation; LY294002: 2-(4-morpholinyl)-8-phenyl-4H-1-benzopyran-4-one; MCAO/r: middle cerebral artery occlusion/reperfusion; TTC: 2,3,5-triphenyltetrazolium chloride; TUNEL: terminal deoxynucleotidyl transferase-mediated dUTP nick end labeling.

In accordance with our experimental design protocol (**Figure 1A**), regional CBF was monitored and documented during the operation using laser speckle flowmetry. Compared with the contralateral side, blood flow decrease by ≥ 70% during the ischemic period, indicating successful vessel occlusion (**Figure 1B** and **E**). Forty-eight hours after the surgery, TTC staining and Nissl staining were used to assess the infarct size resulting from MCAO/r to verify the stability of this experimental model (**Figure 1C** and **D**). The mice exhibited an infarct area of 1.05 ± 0.17 cm^2^, representing approximately 27.72% ± 4.83% of the total area of both hemispheres (3.79 ± 0.21 cm^2^) (**Figure 1F**). This underscores the high repeatability and reliability of the surgical procedures and the MCAO/r model that we used in our experiments. We validated the impact of iTBS on CBF, as well as on infarct size, using laser speckle imaging (**Figure 1G**) and TTC staining (**Figure 1H**), respectively. After 7 days of iTBS, cerebral perfusion on the infarct side increased by 25% (**Figure 1I**), and infarct size decreased by 40%, compared with the MCAO/r group (**Figure 1J**).

### Intermittent theta-burst stimulation upregulates the expression of PI3K/AKT/GSK3β/β-catenin pathway components in the peri-infarct area of mice with cerebral ischemia

**Figure 2:**
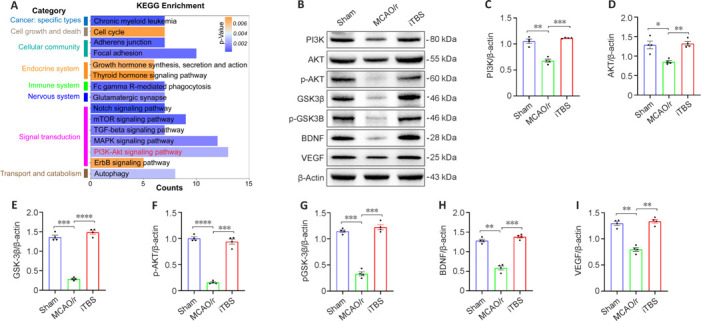
iTBS upregulates the expression of PI3K/AKT/GSK3β/β-catenin pathway components in the peri-infarct area of mice with cerebral ischemia. (A) KEGG pathway analysis showing that iTBS regulates the expression of apoptosis-related signaling pathways, such as the PI3K/AKT pathway. (B) Western blotting showed that iTBS upregulated the expression of PI3K/AKT/GSK3β signaling pathway components, BDNF, and VEGF in the infarct periphery. (C–I) Quantitative analysis of the protein expression shown in B. Data are presented as mean ± SEM (*n* = 4). **P < 0.01, ***P < 0.001 (one-way analysis of variance followed by Tukey’s multiple comparison test). AKT: Protein kinase B; BDNF: brain-derived neurotrophic factor; GSK3β: glycogen synthase kinase-3β; iTBS: intermittent theta burst stimulation; KEGG: Kyoto Encyclopedia of Genes and Genomes; MCAO/r: middle cerebral artery occlusion/reperfusion; p-AKT: phosphorylated-Akt; p-GSK3β: phosphorylated-GSK3β; PI3K: phosphoinositide 3-kinase; VEGF: vascular endothelial growth factor.

**Figure 3:**
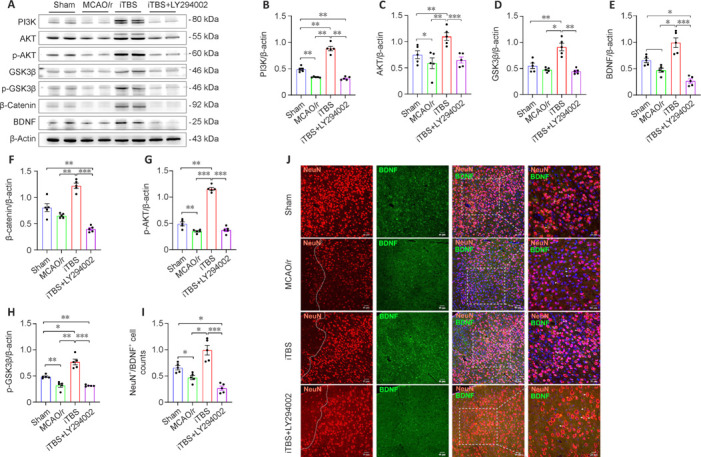
iTBS upregulates the expression of PI3K/AKT/GSK3β/β-catenin signaling pathway components in the peri-infarct area of MCAO/r mice. (A) Immunoblot showing that iTBS upregulated the expression of BDNF/PI3K/AKT/GSK3β/β-catenin signaling pathway components in the peri-infarct area, and LY294002 reversed this effect. (B–H) Quantitative analysis of the protein levels shown in A. (I) Statistical analysis of the numbers of NeuN^+^/BDNF^+^ cells in each group, as seen in J. Data are presented as mean ± SEM (*n* = 4). *P < 0.05, **P < 0.01, ***P < 0.001 (one-way analysis of variance followed by Tukey’s multiple comparison test). (J) Immunofluorescence staining for NeuN (Alexa Fluor 594, red)/BDNF (Alexa Fluor 488, green) showed that iTBS promoted BDNF expression in neurons, and BDNF expression decreased significantly after treatment with LY294002. iTBS significantly promoted neuronal BDNF expression in the peri-infarct area, and LY294002 reversed this effect. Scale bars: 40 μm. AKT: Protein kinase B; BDNF: brain-derived neurotrophic factor; DAPI: 4′,6-diamidino-2-phenylindole; GSK3β: glycogen synthase kinase-3β; iTBS: intermittent theta burst stimulation; LY294002: 2-(4-morpholinyl)-8-phenyl-4H-1-benzopyran-4-one; MCAO/r: middle cerebral artery occlusion/reperfusion; NeuN: neuronal nuclei; p-AKT: phosphorylated-Akt; p-GSK3β: phosphorylated-GSK3β; PI3K: phosphoinositide 3-kinase.

Proteomics analysis and gene enrichment analysis showed that iTBS significantly activated PI3K/AKT signaling in the peri-infarct area of MCAO/r mice (**Figure 2A** and **Additional Figure 1**), and this was confirmed by western blot analysis (**Figure 2B**). In comparison with the MCAO/r group, the protein expression levels of PI3K/AKT/GSK3β signaling pathway components were significantly upregulated in the iTBS treatment group (*P* < 0.001). Moreover, the phosphorylation levels of AKT and GSK3β, which are downstream proteins of PI3K, were significantly elevated in the iTBS treatment group (*P* < 0.001) (**Figure 2C–G**). After 7 days of iTBS intervention, BDNF and VEGF expression levels were significantly upregulated (**Figure 2H** and **I**). Previous studies have shown that BDNF can stimulate the PI3K/AKT/GSK3β/β-catenin signaling pathway in nerve cells, offering protection to both nerves and blood vessels (Gupta et al., 2014; Li et al., 2017). Thus, we hypothesized that iTBS protects nerves and blood vessels after IS by stimulating the BDNF/PI3K/AKT/GSK3β/β-catenin signaling pathway in nerve cells. To test this hypothesis, we employed LY294002, a selective inhibitor of PI3K, to block the PI3K/AKT signaling pathway. The impact of iTBS on the PI3K/AKT/GSK3β/β-catenin signaling pathway was evaluated using western blot analysis and immunofluorescence staining (**Figure 3A**). In comparison with the MCAO/r group, iTBS significantly upregulated the expression of BDNF/PI3K/AKT/GSK3β/β-catenin signaling pathway components in the peri-infarct area of MCAO/r mice (**Figure 3B–I**). Immunofluorescence staining demonstrated that iTBS treatment significantly enhanced p-AKT, p-GSK3β, and BDNF expression in neurons (**Figure 3J** and **Additional Figure 2**), suggesting that iTBS stimulates neuronal PI3K/AKT signaling, which results in GSK3β phosphorylation. Notably, when the PI3K-specific inhibitor LY294002 was employed to block the PI3K/AKT signaling pathway, iTBS-induced upregulation of PI3K/AKT signaling was markedly inhibited, and neuronal p-AKT, p-GSK3β, and BDNF expression levels were reduced. These findings indicate that iTBS regulates the BDNF/PI3K/AKT/GSK3β/β-catenin signaling pathway in neural cells.

### Intermittent theta-burst stimulation reduces neuronal apoptosis in the peri-infarct area via the PI3K/AKT/GSK3β/β-catenin pathway

**Figure 4:**
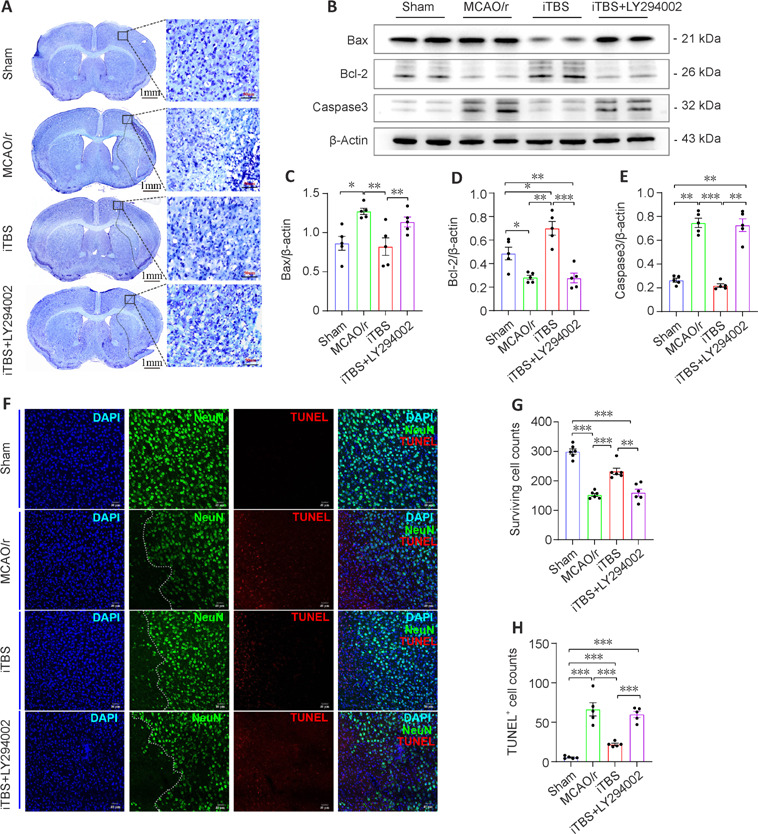
iTBS reduces neuronal apoptosis in the periinfarct area through the PI3K/AKT/GSK3β/β-catenin signaling pathway. (A) Nissl staining was used to detect neuronal injury in the peri-infarct area. Compared with the MCAO/r group, iTBS alleviated neuronal cell damage in the peri-infarct area of mice subjected to cerebral ischemia. (B) Western blotting was used to detect the expression levels of apoptotic proteins in the brain tissue surrounding the infarcted area. (C–E) Quantitative analysis of the protein levels shown in B. (F) NeuN (Alexa Fluor 488, green)/TUNEL (Alexa Fluor 594, red) co-staining was used to detect neuronal apoptosis in the peri-infarct area. Compared with the MCAO/r group, iTBS promoted neuronal survival in the peri-infarct area. (G) Quantitative analysis showed that iTBS significantly promoted neuronal survival in the peri-infarct area, while neuronal injury increased after LY294002 treatment. (H) Quantitative analysis of NeuN/TUNEL staining showed that iTBS significantly reduced neuronal apoptosis in the peri-infarct area. The data are presented as mean ± SEM (*n* = 6). *P < 0.05, **P < 0.01, ***P < 0.001 (one-way analysis of variance followed by Tukey’s multiple comparison test). Bax: BCL2-associated X; BCL2: B-cell lymphoma-2; Caspase3: cysteinyl aspartate specific proteinase; DAPI: 4′,6-diamidino-2-phenylindole; iTBS: intermittent theta burst stimulation; LY294002: 2-(4-morpholinyl)-8-phenyl-4H-1-benzopyran-4-one; MCAO/r: middle cerebral artery occlusion/reperfusion; TUNEL: terminal deoxynucleotidyl transferase-mediated dUTP nick end labeling.

To explore the role of the BDNF/PI3K/AKT/GSK3β/β-catenin signaling pathway in neuronal apoptosis within the peri-infarct area, we employed Nissl staining, western blot analysis, TUNEL, and NeuN immunofluorescence staining. Nissl staining revealed disorganized nerve cells, irregular morphology, and widespread Nissl body shrinkage or loss in the peri-infarct area of the MCAO/r group, compared with the Sham and iTBS groups (**Figure 4A**). iTBS intervention significantly increased the number of surviving nerve cells in the peri-infarct area (*P* < 0.001), whereas LY294002 treatment significantly reduced cell survival (*P* < 0.01). Western blot analysis indicated that Bax and Caspase 3 expression levels were significantly reduced (*P* < 0.01), while Bcl-2 expression was markedly elevated in the iTBS treatment group compared with the MCAO/r group. LY294002 reversed the effects of iTBS on apoptotic proteins expression (**Figure 4B–E**). TUNEL and NeuN immunofluorescence staining showed that, compared with the Sham group, the number of apoptotic neurons in the peri-infarct area of mice in the MCAO/r group was significantly increased, while iTBS significantly decreased the number of apoptotic neurons (*P* < 0.001; **Figure 4F**). In comparison with the iTBS group, the number of apoptotic neurons in the LY294002 group was significantly higher (*P* < 0.001; **Figure 4G** and **H**). These results show that iTBS reduces neuronal apoptosis in the peri-infarct region of MCAO/r mice by modulating BDNF/PI3K/AKT/GSK3β/β-catenin signaling.

### Intermittent theta-burst stimulation promotes motor function recovery in mice with ischemic injury via the PI3K/AKT/GSK3β/β-catenin pathway

**Figure 5:**
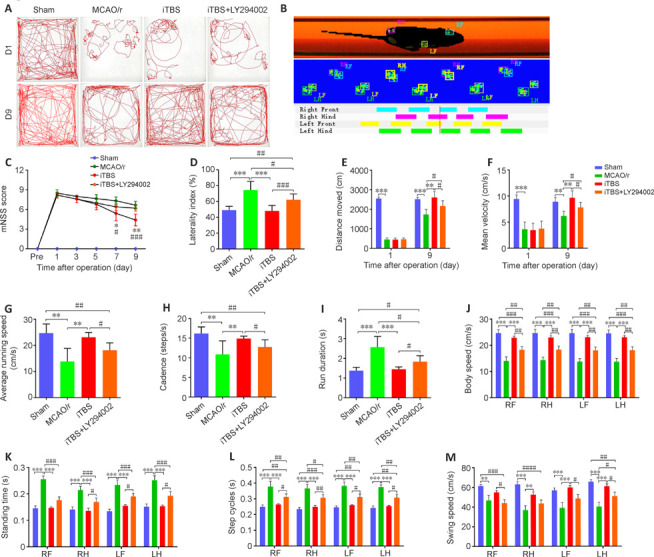
iTBS promotes neurological and motor function recovery in MCAO/r mice through the PI3K/AKT/GSK3β/β-catenin signaling pathway. (A) The open field test was used to assess mouse running trajectories in an open field. Compared with the MCAO/r group, iTBS increased the distance traveled and the average running speed of mice in the open field. (B) Catwalk gait analysis was used to assess mouse gait. (C–M) In comparison with the MCAO/r and LY294002 groups, iTBS significantly reduced the mNSS score (C) and the lateralization index of the bilateral forelimbs (D), increased the distance (E) and average running speed (F) in the open field, increased average walking speed (G), gait frequency (H) limb movement speed (J) and body swing speed (M) in the runway test, and reduced the walking time (I), standing time (K), and gait cycle (L) in mice subjected to ischemia. Data are presented as mean ± SEM (*n* = 6). *P < 0.05, **P < 0.01, ***P < 0.001 (one-way analysis of variance followed by Tukey’s multiple comparison test [D–M] and two-way analysis of variance followed by Bonferroni’s multiple comparison test [C]). AKT: Protein kinase B; GSK3β: glycogen synthase kinase-3β; iTBS: intermittent theta burst stimulation; LF: left front; LH: left hand; LY294002: 2-(4-morpholinyl)-8-phenyl-4H-1-benzopyran-4-one; MCAO/r: middle cerebral artery occlusion/reperfusion; mNSS: modified Neurologic Severity Scores; PI3K: phosphoinositide 3-kinase; RF: right front; RH: right hand.

Given our observation that iTBS reduced neuronal apoptosis, we next investigated the effect of iTBS on the behavior of MCAO/r mice. To do this, we used the mNSS scale to assess the neurological deficits of mice before the operation and on days 1, 3, 5, 7, and 9 post-operation (**Figure 5C**). On day 5 of iTBS treatment, the mNSS score for MCAO/r mice was significantly lower (*P* < 0.05) than both the MCAO/r group and the inhibitor intervention group. Additionally, neurological recovery in the MCAO/r mice was notably slowed by inhibitor treatment. The cylinder test was used to detect the laterality index of the bilateral forelimbs of the mice in each group (**Figure 5D**). In comparison with the Sham group, the laterality index of the mice in the MCAO/r group was significantly increased (*P* < 0.001), indicating that the mice preferred to use their left front paw when exploring inside the cylinder. After 7 days of iTBS treatment, the laterality index of the mice subjected to cerebral ischemia was significantly reduced (*P* < 0.001), reaching levels close to those of mice in the Sham group. The laterality index of the mice in the inhibitor intervention group was greater than that of the mice in the iTBS treatment group (*P* < 0.001) and lower than that of the mice in the MCAO/r group (*P* < 0.05). These results indicate that iTBS enhances recovery of neurological function and improves bilateral forelimb coordination in MCAO/r mice through the BDNF/PI3K/AKT/GSK3β/β-catenin signaling pathway.

Compared with the Sham group, the moving distance and speed of the mice in the open field were significantly reduced on the first day following MCAO/r surgery (*P* < 0.0001) (**Figure 5A**). After 7 days of iTBS treatment, compared with the MCAO/r group, both the moving distance and average speed of the mice subjected to cerebral ischemia in the open field were significantly increased compared with the MCAO/r group (*P* < 0.001). Treatment with the inhibitor significantly decreased the movement distance and running speed of the MCAO/r mice (*P* < 0.05; **Figure 5E** and **F**). Catwalk gait analysis (**Figure 5B**) showed that mice in the MCAO/r group exhibited significantly reduced walking speed and limb swing speed, shortened step length, decreased step frequency, and significantly prolonged standing time in the runway compared with the Sham group (**Figure 5G–M**). After 7 days of iTBS treatment, the standing time of MCAO/r mice on the runway was significantly reduced, and step frequency, limb movement speed, and step length were all significantly increased (*P* < 0.01). By contrast, compared with the iTBS group, treatment with the inhibitor increased the standing time of mice on the runway and decreased the step frequency, limb movement speed, and step length of the mice (*P* < 0.05). These results show that iTBS improves motor and gait function in mice subjected to MCAO/r via the BDNF/PI3K/AKT/GSK3β/β-catenin signaling pathway.

### Intermittent theta-burst stimulation modulates microglial polarization via the PI3K/AKT/GSK3β pathway, thereby improving the inflammatory microenvironment

**Figure 6:**
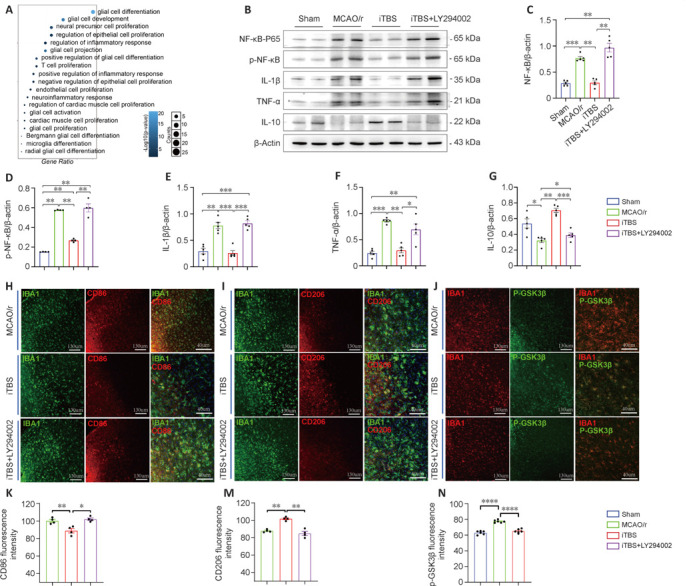
iTBS mediate microglial polarization through the PI3K/AKT/GSK3β signaling pathway, thereby improving the inflammatory microenvironment in the peri-infarct area. (A) Gene Ontology analysis was performed on significantly differentially expressed genes, and the bar plot illustrates that these genes were significantly enriched in functional terms related to glial cell proliferation, differentiation, and neuroinflammation regulation. (B) Western blotting was used to detect the expression of inflammatory factors (NF-κB, p-NF-κB, IL-1β, TNF-α, and IL-10) in peri-infarct brain tissue. (C–G) Quantitative analysis of the protein levels shown in B. (H, I) Immunofluorescence staining was performed to detect the microglial cell marker IBA1 (Alexa Fluor 488, green), as well as CD86 (Alexa Fluor 594, red) and CD206 (Alexa Fluor 594, red), in the peri-infarct area. Compared with the MCAO/r group, iTBS enhanced CD206 expression in microglia within the peri-infarct area while reducing the expression of CD86. Scale bars: 130 μm and 40 μm. (J) The immunofluorescence staining detection showed the microglial cell marker IBA1 (Alexa Fluor 594, red) co-labeled with p-GSK3β (Alexa Fluor 488, green) in the peri-infarct area. Compared with the MCAO/r group, iTBS induced high levels of p-GSK3β expression in microglia. Scale bars: 130 μm and 40 μm. (K–N) Statistical analysis of the fluorescence expression intensity data shown in H–J. The data are presented as mean ± SEM (*n* = 4–6). *P < 0.05, **P < 0.01, ***P < 0.001 (one-way analysis of variance followed by Tukey’s multiple comparison test). AKT: Protein kinase B; IBA1: ionized calcium binding adaptor molecule 1; GSK3β: glycogen synthase kinase-3β; IL-10: interleukin-10; IL-1β: interleukin-1β; iTBS: intermittent theta burst stimulation; LY294002: 2-(4-morpholinyl)-8-phenyl-4H-1-benzopyran-4-one; MCAO/r: middle cerebral artery occlusion/reperfusion; NF-κB: nuclear factor kappa-B; P-NF-κB: phosphorylated-NF-κB; PI3K: phosphoinositide 3-kinase; TNF-α: tumor necrosis factor-α; ZO-1: zonula occludens protein 1.

Gene Ontology functional enrichment analysis revealed that iTBS significantly altered the expression levels of proteins enriched in terms related to neuronal survival, glial cell proliferation, differentiation, and inflammation. These functional terms are strongly linked to the PI3K/AKT pathway (**Figure 6A**). Western blotting (**Figure 6B**) showed that, compared with the MCAO/r and iTBS + LY294002 groups, the iTBS intervention group exhibited significantly lower levels of pro-inflammatory factors (NF-κB, p-NF-κB, IL-1β, and TNF-α) (*P* < 0.01) and elevated levels of the anti-inflammatory factor IL-10 (*P* < 0.01) (**Figure 6C–G**). Immunofluorescence staining revealed that iTBS significantly elevated p-GSK3β (*P* < 0.001; **Figure 6J**) and M2 microglial marker CD206 expression levels while reducing M1 marker CD86 expression (*P* < 0.01; **Figure 6H** and **I**). LY294002 treatment significantly decreased p-GSK3β and CD206 expression (*P* < 0.01) and increased CD86 expression (*P* < 0.05; **Figure 6K–N**). These findings indicate that iTBS mediates microglial phenotypic transformation via the PI3K/AKT/GSK3β pathway, thereby improving the local inflammatory microenvironment.

### Intermittent theta-burst stimulation may help regulate astrocyte polarization in the peri-infarct area through the PI3K/AKT/GSK3β pathway

After ischemic brain injury, reactive astrocytes proliferate in the peri-infarct area and gradually form a glial scar, which has both beneficial and harmful effects on neurons (Han et al., 2022). In addition, astrocytes are necessary for neurovascular remodeling (Williamson et al., 2021). Our results showed that iTBS promoted BDNF expression in neurons (**Figure 3B**) and astrocytes (**Additional Figure 3**) in the peri-infarct area via the PI3K/AKT signaling pathway. Additionally, A2 astrocytes serve as a significant source of BDNF during IS (Lessmann and Brigadski, 2009), suggesting that iTBS may mediate astrocyte polarization in mice subjected to MCAO/r through the PI3K/AKT pathway, which can affect stroke outcomes.

**Figure 7:**
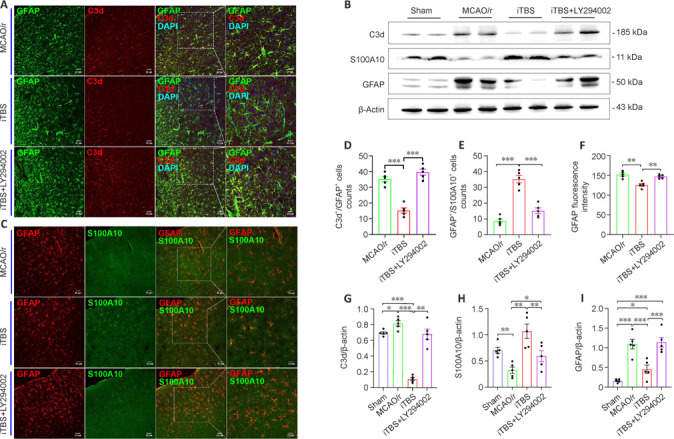
iTBS promotes polarization of astrocytes in the peri-infarct area towards the A2 phenotype via the PI3K/AKT/GSK3β/β-catenin signaling pathway. (A) Immunofluorescence staining for GFAP (Alexa Fluor 488, green) and C3d (Alexa Fluor 594, red) in brain tissue. Compared with the MCAO/r group, iTBS significantly reduced C3d expression in astrocytes. Scale bars: 40 μm. (B) Western blot analysis of C3d, S100A10, and GFAP expression in brain tissue. (C) Immunofluorescence staining for GFAP (Alexa Fluor 594, red) and S100A10 (Alexa Fluor 488, green) in brain tissue. Compared with the MCAO/r group, iTBS induced high levels of S100A10 expression in astrocytes. Scale bars: 40 μm. (D) Statistical analysis of the numbers of GFAP^+^/C3d^+^ cells in each experimental group. (E) Statistical analysis of the numbers of GFAP^+^/S100A10^+^ cells in C. (F) Statistical analysis of the GFAP immunofluorescence intensity data shown in C. (G–I) Quantitative analysis of C3d, S100A10, and GFAP expression in the peri-infarct area, as shown in A and B. The data are presented as mean ± SEM (*n* = 5). *P < 0.05, **P < 0.01, ***P < 0.001 (one-way analysis of variance followed by Tukey’s multiple comparison test). AKT: Protein kinase B; DAPI: 4′,6-diamidino-2-phenylindole; GFAP: glial fibrillary acidic protein; GSK3β: glycogen synthase kinase-3β; iTBS: intermittent theta burst stimulation; LY294002: 2-(4-morpholinyl)-8-phenyl-4H-1-benzopyran-4-one; MCAO/r: middle cerebral artery occlusion/reperfusion; PI3K: phosphoinositide 3-kinase.

We used immunofluorescence staining and western blotting to assess expression of the A1 astrocyte marker C3d and the A2 astrocyte marker S100A10 in the peri-infarct area in each experimental group (**Figure 7A–I**). Compared with the MCAO/r group, C3d and GFAP expression levels in astrocytes were significantly reduced in the peri-infarct area in the iTBS treatment group, while S100A10 expression was significantly increased. In addition, western blotting indicated that iTBS increased S100A10 expression and inhibited C3d and GFAP expression in peri-infarct brain tissue, indicating that iTBS may help regulate astrocyte polarization in the peri-infarct area through the PI3K/AKT/GSK3β pathway.

### Intermittent theta-burst stimulation strengthens the blood–brain barrier and promotes A2 astrocyte coupling with blood vessels in the peri-infarct area via the PI3K/AKT/GSK3β pathway

**Figure 8:**
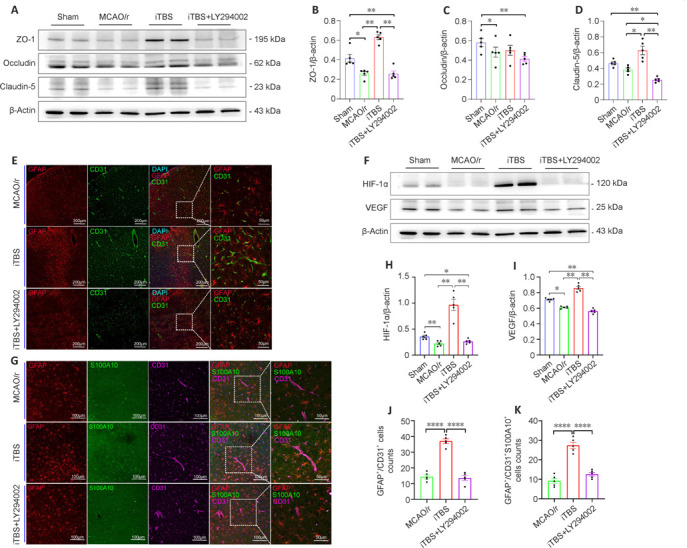
iTBS strengthens the blood–brain barrier and promotes coupling of A2 astrocytes with blood vessels in the peri-infarct area via the PI3K/AKT/GSK3β pathway. (A) Western blotting was used to detect the expression of tight junction proteins, including ZO-1, claudin-5, and occludin, in peri-infarct brain tissue. (B–D) Quantitative analysis of ZO-1, claudin-5, and occludin expression, as detected by western blotting. (E) Immunofluorescence staining for GFAP (Alexa Fluor 594, red) and CD31 (Alexa Fluor 647, pink). Compared with the MCAO/r group, iTBS increased interactions between astrocytes (GFAP) and blood vessels (CD31). Scale bars: 50 μm. (F) Western blot analysis of HIF-1α and VEGF expression in the peri-infarct area. (G) Immunofluorescence staining for GFAP (Alexa Fluor 594, red), S100A10 (Alexa Fluor 488, green), and CD31 (Alexa Fluor 594, pink). iTBS enhanced interactions between A2-type astrocytes (GFAP) and blood vessels (CD31). Scale bars: 50 μm. (H, I) Quantitative analysis of HIF-1α and VEGF expression, as detected by western blotting. (J) Statistical analysis of the numbers of GFAP^+^/CD31^+^ cells shown in E. (K) Statistical analysis of the numbers of GFAP^+^/CD31^+^/S100A10^+^ cells shown in G. The data are presented as mean ± SEM (*n* = 5). *P < 0.05, **P < 0.01, ***P < 0.001 (one-way analysis of variance followed by Tukey’s multiple comparison test). AKT: Protein kinase B; DAPI: 4′,6-diamidino-2-phenylindole; GFAP: glial fibrillary acidic protein; DAPI: 4′,6-diamidino-2-phenylindole; HIF-1α: hypoxia-inducible factor 1α; iTBS: intermittent theta burst stimulation; LY294002: 2-(4-morpholinyl)-8-phenyl-4H-1-benzopyran-4-one; MCAO/r: middle cerebral artery occlusion/reperfusion; PI3K: phosphoinositide 3-kinase; VEGF: vascular endothelial growth factor.

Previous studies have reported that the PI3K/AKT/GSK3β/β-catenin signaling pathway can induce astrocyte polarization to reduce inflammation (Wei et al., 2019) and promote angiogenesis in IS (Zhan et al., 2020). However, it is still uncertain whether iTBS can influence astrocyte polarization through the PI3K/AKT/GSK3β/β-catenin pathway to maintain NVU integrity. Western blot analysis revealed that iTBS significantly increased the expression of tight junction proteins, including ZO-1, claudin-5, and occludin (**Figure 8A–D** and **Additional Figure 4**) and vascular proliferation indicators, such as VEGF and HIF-1α (**Figure 8F**, **H**, and **I**). Notably, the protective effect of iTBS on blood vessels was blocked by treatment with LY294002. Immunofluorescence co-staining for GFAP and CD31 (**Figure 8E**) showed that, in comparison with the MCAO/r group, iTBS treatment notably enhanced co-localization of astrocyte processes with blood vessels, and that this co-localization correlated positively with increased blood vessel density and continuity in the peri-infarct area. Furthermore, CD31/GFAP/S100A10 immunofluorescence co-staining revealed that iTBS strongly increased S100A10 expression in astrocytes that were mutually coupled with blood vessels (**Figure 8G**), to levels surpassing those seen in both the MCAO/r group and the inhibitor intervention group (**Figure 8J** and **K**). These findings indicate that iTBS may modulate astrocyte polarization and enhancing coupling between astrocytes and blood vessels, potentially through the PI3K/AKT/GSK3β/β-catenin signaling pathway, thereby improving NVU structural integrity.

## Discussion

In this study, we examined the neuroprotective and vasoprotective effects of iTBS on the peri-infarct area in a mouse model of ischemic brain injury. Our experimental results demonstrated that applying iTBS to the ipsilateral hemisphere during the acute phase of stroke significantly inhibited the neuronal apoptosis induced by cerebral ischemia, improved CBF, and enhanced motor function in ischemic mice. Furthermore, proteomics analysis and subsequent experiments revealed that iTBS promoted microglial proliferation and transformation to the anti-inflammatory M2 phenotype via the PI3K/AKT/GSK3β signaling pathway. In addition, iTBS facilitated coupling between A2 astrocytes and blood vessels, contributing to improved neurovascular coupling after ischemic brain injury.

rTMS, a non-invasive neuromodulation technique, is widely employed in treating neurodegenerative diseases such as stroke, depression, and Parkinson’s disease (Uzair et al., 2022; Yu et al., 2023; Wang et al., 2024). An earlier study showed that high-frequency rTMS (10 Hz) is more effective than low-frequency rTMS (1 Hz) in blocking apoptosis, regulating inflammation, and improving neurological and motor function (Sasaki et al., 2013). Our findings similarly reveal that iTBS in the ipsilateral hemisphere promotes BDNF release, reduces neuronal apoptosis in the peri-infarct region, and enhances recovery in ischemic mice. Although the neuroprotective effects of rTMS are well established, its influence on non-neuronal cells, including microglia and astrocytes, and the underlying molecular mechanisms is less well explored.

With the NVU concept gaining attention, stroke rehabilitation has shifted from neuroprotection to neurovascular protection (Iadecola, 2017). Unlike traditional therapies, rTMS offers precise neurovascular modulation. Our laser speckle flowmetry results revealed a 20% increase in blood flow in the infarcted hemisphere after 7 days of iTBS, suggesting improved neurovascular coupling. Astrocytes, which are key to NVU function, bridge neurons and blood vessels, reducing excitotoxicity, protecting neurons, and regulating blood flow (Attwell et al., 2010). Prior research indicates that high-frequency rTMS enhances astrocyte proliferation (Bai et al., 2023), modulates inflammation, and maintains the blood–brain barrier (Zong et al., 2020). Our study showed that iTBS promotes anti-inflammatory A2 astrocyte transformation, reduces inflammation, strengthens the blood–brain barrier, and enhances astrocyte–blood vessel coupling, explaining its role in improving perfusion post–ischemic injury. Animal studies have shown that rTMS significantly alleviates acute brain edema in a rat model of hemorrhagic stroke and facilitates the recovery of neurological function (Cui et al., 2019). Therefore, rTMS could help mitigate the acute reperfusion injury associated with stroke and improve long-term outcomes.

After ischemic brain injury, interactions between microglia and astrocytes are crucial for NVU remodeling (Jha et al., 2019; Liu et al., 2020a). Microglia, as the brain’s primary immune cells, are rapidly activated during the acute phase of stroke (Zhao et al., 2017), while astrocyte proliferation and transformation are regulated by microglia-mediated inflammation. M1 microglia are the main source of IL-1β, which induces astrocyte adoption of the pro-inflammatory A1 phenotype (Liu et al., 2020a). Thus, early pro-inflammatory signals from microglia guide astrocyte activation and phenotypic transformation (Ferreira et al., 2024). We previously found that iTBS downregulates members of the Toll-like receptor 4/NF-κB/NOD-like receptor family and components of the pyrin domain containing 3 pathway, thereby promoting M2 microglia transformation and improving functional recovery in ischemic mice (Luo et al., 2022b). However, iTBS does not exhibit neuroprotective effects when microglia are depleted, indicating that microglia are essential for its protective role (Luo et al., 2022b). Whether rTMS affects astrocyte proliferation and transformation directly or indirectly via microglial regulation requires further investigation.

Proteomics analysis showed significant modulation of the PI3K/AKT and NF-κB pathways by iTBS. Earlier studies have suggested that PI3K/AKT activation inhibits pro-inflammatory M1 microglia and promotes anti-inflammatory A2 astrocyte transformation (Xu et al., 2018; Wei et al., 2019). We found that iTBS-induced activation of the PI3K/AKT/GSK3β pathway in the peri-infarct region enhances neuron survival, vascular protection, and blood–brain barrier integrity and reduces inflammation, thereby improving motor function. The neuroprotective effects of iTBS were reduced by treatment with the PI3K inhibitor LY294002, suggesting that the PI3K/AKT/GSK3β pathway is crucial for iTBS’s neuroprotective effects. In addition, HDAC3, a neurotoxic protein, is expressed at high levels after ischemic brain injury (Liao et al., 2020) and is linked to microglial activation and pro-inflammatory gene expression, regulated by AKT and GSK3β (Bardai and D’Mello, 2011). Studies show that reducing HDAC3 expression in microglia inhibits their transformation into the pro-inflammatory M1 phenotype without affecting M2 microglia (Liao et al., 2020; Zhang et al., 2024). Our western blot results indicated that LY294002 treatment increased the expression of pro-inflammatory mediators (IL-1β, and TNF-α) and decreased IL-10 expression, suggesting involvement of the PI3K/AKT/GSK3β pathway in microglial proliferation and pro-inflammatory mediator release. Immunofluorescence revealed high levels of p-GSK3β expression in microglia after iTBS, while treatment with LY294002 reduced these levels. These findings suggest that iTBS regulates microglial proliferation through the PI3K/AKT/GSK3β pathway, thereby influencing astrocyte activation.

This study had some limitations. First, while we observed that rTMS can modulate CBF, future research could combine rTMS with two-photon *in vivo* imaging to monitor its effects on neurovascular coupling in the peri-infarct region following ischemic brain injury. Additionally, further investigation is required to confirm the impact of early high frequency rTMS on HDAC3 expression in microglia during the acute phase of ischemic brain injury and to clarify the mechanisms by which rTMS regulates microglial proliferation.

In conclusion, our findings demonstrate that iTBS regulates microglial and astrocyte polarization in the peri-infarct zone, promotes neurovascular unit remodeling, and improves neurological and motor function in mice subjected to MCAO/r via the PI3K/AKT/GSK3β and NF-κB signaling pathways.

## Additional files:

***Additional Figure 1:*** *Significantly differentially expressed proteins.*

Additional Figure 1 Significantly differentially expressed proteins.
The volcano plot illustrates the significantly differentially expressed proteins, with red indicating significantly upregulated proteins and blue indicating significantly downregulated proteins. These proteins were subsequently subjected to functional and pathway enrichment analyses.

***Additional Figure 2:*** *iTBS promotes the expression of p-AKT and p-GSK3β in neurons in the peri-infarct area of mice.*

Additional Figure 2 iTBS promotes neuronal p-AKT and p-GSK3β expression in the peri-infarct area of mice.
(A) Immunofluorescence staining for NeuN (Alexa Fluor594, red) and p-AKT (Alexa Fluor 488, green). Compared with the MCAO/r group, iTBS promoted neuronal p-AKT expression in the peri-infarct area. Scale bars: 40 μm. (B) Immunofluorescence staining for NeuN (Alexa Fluor 594, red) and p-GSK3β (Alexa Fluor488, green). Compared with the MCAO/r group, iTBS promoted neuronal p-GSK3β expression in the peri-infarct area. Scale bars: 40 μm. (C) Quantitative analysis of the p-AKT immunofluorescence intensity data shown in A. (D) Quantitative analysis of the p-GSK3β immunofluorescence intensity data shown in B. The data are presented as mean ± SEM (n = 4). ^*^P < 0.05, ^**^P < 0.01, ^***^P < 0.001 (one-way analysis of variance followed by Tukey’s multiple comparison test). Akt: Protein kinase B; DAPI: 4ʹ,6-diamidino-2-phenylindole; GSK3β: glycogen synthase kinase-3β; iTBS: intermittent theta burst stimulation; LY294002: 2-(4-morpholinyl)-8-phenyl-4H-1-benzopyran-4-one; MCAO/r: middle cerebral artery occlusion/reperfusion; NeuN: neuronal nuclei; p-AKT: phosphorylated-Akt; p-GSK3β: phosphorylated-GSK3β.

***Additional Figure 3:*** *iTBS promotes the expression of BDNF in astrocytes in the peri-infarct area of mice.*

Additional Figure 3 iTBS promotes BDNF expression in astrocytes in the peri-infarct area of mice.
(A) Immunofluorescence staining for GFAP (Alexa Fluor 594, red) and BDNF (Alexa Fluor 488, green). Compared with the MCAO/r group, iTBS promoted BDNF expression in astrocytes in the peri-infarct area. Scale bars: 130 μm and 40 μm. (B) Quantitative analysis of the GFAP^+^/BDNF^+^ colocalization shown in A. The data are presented as mean ± SEM (n = 5). ^**^P < 0.01, ^***^P < 0.001 (one-way analysis of variance followed by Tukey’s multiple comparison test). BDNF: Brain-derived neurotrophic factor; GFAP: glial fibrillary acidic protein; iTBS: intermittent theta burst stimulation; LY294002: 2-(4-morpholinyl)-8-phenyl-4H-1-benzopyran-4-one; MCAO/r: middle cerebral artery occlusion/reperfusion.

***Additional Figure 4:*** *iTBS promotes the expression of Claudin5 in astrocytes in the peri-infarct area* *of mice.*

Additional Figure 4 iTBS promotes claudin-5 expression in astrocytes in the peri-infarct area.
(A) Immunofluorescence staining for GFAP (Alexa Fluor 594, red) and claudin-5 (Alexa Fluor 488, green). Compared with the MCAO/r group, iTBS promoted claudin-5 expression in astrocytes in the peri-infarct area. Scale bars: 130 μm and 40 μm. (B) Quantitative analysis of the GFAP^+^/Claudin-5^+^ colocalization shown in A. The data are presented as mean ± SEM (n = 5). ^***^P < 0.001 as (one-way analysis of variance followed by Tukey’s multiple comparison test). GFAP: Glial fibrillary acidic protein; iTBS: intermittent theta burst stimulation; LY294002: 2-(4-morpholinyl)-8-phenyl-4H-1-benzopyran-4-one; MCAO/r: middle cerebral artery occlusion/reperfusion.

## Data Availability

*All data relevant to the study are included in the article or uploaded as Additional files.*
